# 

**Published:** 1843-04

**Authors:** 


### THE
### WESTERN JOURNAL
### OF
### MEDICINE AND SURGERY:

EDITED BY

DANIEL DRAKE, M. D.

AND

LUNSFORD P. YANDELL, M. D.

PROFESSORS IN THE LOUISVILLE MEDICAL INSTITUTE,

AND

THOMAS W. COLESCOTT, M. D.

---

**NO. XL.—APRIL, 1843.**

Vol. VII.—No. IV.

---

LOUISVILLE, KY.

PUBLISHED MONTHLY, BY PRENTICE AND WEISSINGER,

PROPRIETORS AND PRINTERS.

1843.

*This Number contains 4 sheets—Postage under 100 miles 6 cents, over 100 miles 10 cents*

